# Is reiki or prayer effective in relieving pain during hospitalization for cesarean? A systematic review and meta-analysis of randomized controlled trials: O *reiki* ou a oração são efetivos no alívio da dor durante a internação da cesariana? Uma revisão sistemática e metanálise de ensaios clínicos randomizados

**DOI:** 10.1590/1516-3180.2016.0267031116

**Published:** 2017-04-20

**Authors:** Guilherme Augusto Rago Ferraz, Meline Rosseto Kron Rodrigues, Silvana Andrea Molina Lima, Marcelo Aparecido Ferraz Lima, Gabriela Lopes Maia, Carlos Alberto Pilan, Michelle Sako Omodei, Ana Cláudia Molina, Regina El Dib, Marilza Vieira Cunha Rudge

**Affiliations:** I MSc. PhD’s Student, Postgraduate Program on Gynecology, Obstetrics and Mastology, Universidade Estadual Paulista (UNESP), Botucatu (SP), Brazil.; II PhD. Assistant Professor, Department of Nursing, Universidade Estadual Paulista (UNESP), Botucatu (SP), Brazil.; III BA. Master’s Student, Postgraduate Program on Public Health, Universidade Estadual Paulista (UNESP), Botucatu (SP), Brazil.; IV Undergraduate Nursing Student, Faculdade Marechal Rondon (FMR), São Manoel (SP), Brazil.; V Undergraduate Medical Student, Universidade Federal de Minas Gerais (UFMG), Belo Horizonte (MG), Brazil.; VI MD. Physician. Department of Gynecology and Obstetrics, Universidade Estadual Paulista (UNESP), Botucatu (SP), Brazil.; VII PhD. Nurse, Botucatu Outpatient Clinics, Municipal Authority of Botucatu, Botucatu (SP), Brazil.; VIII PhD. Assistant Professor, Institute of Science and Technology, Department of Biosciences and Oral Diagnosis, Universidade Estadual Paulista (UNESP), São José dos Campos (SP), Brazil, and Research collaborator, Institute of Urology, McMaster University, Hamilton, Ontario, Canada.; IX MD, PhD. Titular Professor, Department of Gynecology and Obstetrics, Universidade Estadual Paulista (UNESP), Botucatu (SP), Brazil.

**Keywords:** Spirituality, Delivery, obstetric, Complementary therapies, Risk factors, Review

## Abstract

**CONTEXT AND OBJECTIVE:**

This systematic review compared reiki and prayer with drug use for relieving pain during hospitalization for cesarean, given that the popularity of integrative medicine and spiritual healing has been increasing. It had the aim of evaluating whether reiki or prayer is effective in relieving pain during cesarean section.

**DESIGN AND SETTING:**

Systematic review with meta-analysis conducted at Botucatu Medical School, UNESP, São Paulo, Brazil.

**METHODS:**

The following databases were searched up to March 2016: MEDLINE, Embase, LILACS and CENTRAL. Randomized controlled trials published in English or Portuguese were included in the review. Two reviewers independently screened eligible articles, extracted data and assessed the risk of bias. A GRADE table was produced to evaluate the risk of bias.

**RESULTS:**

There was evidence with a high risk of bias showing a statistically significant decrease in pain score through use of reiki and prayer, in relation to the protocol group: mean difference = -1.68; 95% confidence interval: -1.92 to -1.43; P < 0.00001; I^2^ = 92%. Furthermore, there was no statistically significant difference in heart rate or systolic or diastolic blood pressure.

**CONCLUSION:**

Evidence with a high risk of bias suggested that reiki and prayer meditation might be associated with pain reduction.

## RESUMO

**CONTEXTO E OBJETIVO:**

Esta revisão sistemática comparou o *reiki* e a oração ao uso de medicamentos, a fim de aliviar a dor durante a internação para cesariana, visto que há um aumento na popularidade da medicina integrativa e cura espiritual. Esta revisão teve como objetivo avaliar se o *reiki* ou oração são eficazes no alívio da dor durante a cesariana.

**TIPO DE ESTUDO E LOCAL:**

Revisão sistemática com metanálise realizada na Faculdade de Medicina de Botucatu UNESP, São Paulo, Brasil.

**MÉTODOS:**

As seguintes bases de dados foram pesquisadas até março de 2016: MEDLINE, Embase, LILACS e CENTRAL. Nesse sentido, foram incluídos ensaios clínicos randomizados publicados em inglês e português. Dois revisores rastrearam independentemente artigos elegíveis, extraíram dados e avaliaram o risco de viés. A tabela GRADE foi realizada para avaliar o risco de viés.

**RESULTADOS:**

Evidências com alto índice de viés encontraram uma diminuição estatisticamente significativa na redução da dor (diferença média = -1,68; intervalo de confiança de 95%: -1,92 a -1,43; P < 0,00001; I^2^ = 92%), com o uso de *reiki* e oração comparado com o grupo protocolar. Além disso, não houve diferença estatisticamente significativa na frequência cardíaca, pressão arterial sistólica e diastólica.

**CONCLUSÃO:**

Evidência com alto risco de viés sugeriu que *reiki* e meditação oração podem ser associadas com a redução da dor.

## INTRODUCTION

Complementary therapies have been practiced since ancient times, but there is still little scientific evidence on their real efficiency. Most of these therapies originated from oriental cultures, such as in India with Ayurveda treatments; China with acupuncture and moxibustion therapies; and Japan with reiki therapy. Moreover, complementary therapies are implemented both alone and alongside conventional medicine. Thus, complementary therapies tend to take a holistic approach in order to treat the entire person, i.e. body, mind and soul. In other words, they use a comprehensive set of techniques, such as meditation, body therapies, energy manipulation, art and music therapy, dietary therapy and other procedures that involve healthcare, according to the National Center for Complementary and Alternative Medicine.^1^^,^^2^^,^^3^^,^^4^^,^^5^

Reiki is an ancient Japanese form of hands-on healing. The term comes from combining two Japanese words: rei, a universal spirit; and ki, meaning universal life energy.^1^ Despite being a Japanese form of healing, use of reiki has already spread worldwide. It is mainly used for pain relief.^2^ Additionally, prayer meditation is also considered to be an adjunctive therapy involving a non-invasive method with a low-cost procedure.^5^ Thus, it improves psychological, social, spiritual and physical health by means of nourishing the environment through peacefulness and mindfulness.^6^^,^^7^

A previous systematic review of clinical trials^2^ compared reiki therapy with the usual care or with placebo among women undergoing breast biopsy, women with abdominal hysterectomies, cancer patients, individuals with depression, and chronically ill patients. However, that review seemed to have serious limitations with regard to its methodological aspects. For example, it presented a variety of conditions, i.e. 12 articles were included and therefore 12 different types of conditions, but there were no data on pregnant women. In other words, the review was quite generalist. Moreover, it did not use the GRADE approach to rate the quality of scientific evidence. Consequently, the review was unable to provide any conclusion about the effectiveness of reiki and the suggestion made was that new studies on this topic would be necessary.

In the literature, a few studies^1^^,^^2^^,^^3^^,^^4^^,^^5^^,^^6^^,^^7^^,^^8^^,^^9^^,^^10^^,^^11^ have reported that spirituality and complementary therapies have provided improvements regarding quality of life and benefits in relation to several health conditions.^6^^,^^7^^,^^8^ Moreover, it has been suggested that non-pharmacological practices could be considered in order to reduce excessive use of allopathic medication in obstetrics and consequently to reduce the costs of care.

## OBJECTIVE

The aim of this systematic review of randomized controlled trials (RCTs) was to evaluate whether reiki or prayer meditation is effective for controlling pain among women undergoing cesarean section.

## METHODS

The Cochrane Handbook for Intervention Reviews^12^ guided our choice of methods. Our reporting adhered to the Preferred Reporting Items for Systematic Reviews and Meta-analysis (PRISMA) statement.^13^

### Eligibility criteria

We included RCTs or quasi-RCTs that compared reiki therapy and prayer meditation with the usual care among pregnant women undergoing cesarean section, including any of the following maternal outcomes before and after receiving the intervention or usual care: pain control; heart rate; diastolic and systolic blood pressure; or medication intake. Furthermore, a single study^9^ recorded postpartum physical activities through the Milestone questionnaire.

### Data source and searches

Pertinent literature was identified through MEDLINE (from 1966 to March 2016); Embase (from 1980 to March 2016); LILACS (from 1982 to March 2016); and Cochrane controlled trials (CENTRAL) (up to March 2016), using the terms spirituality, reiki, prayer, cesarean and labor pain (Table 1). The data-gathering was restricted to Portuguese and English-language studies. There were no publication status restrictions. A review of relevant references in previous systematic review articles^1^^,^^2^ and primary studies^3^^,^^4^^,^^5^^,^^6^^,^^7^^,^^8^^,^^9^^,^^10^^,^^11^ was conducted.

**Table 1:**
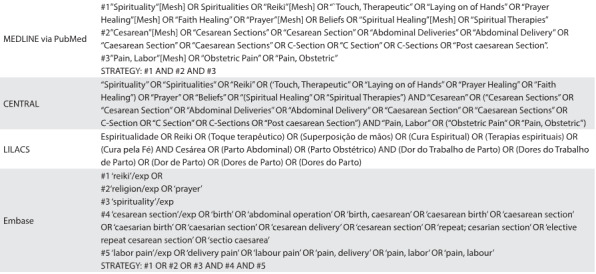
Search strategies used in MEDLINE (via PubMed), CENTRAL, LILACS and Embase

### Selection of studies 

Randomized controlled trials or quasi-RCTs published in English or Portuguese were included. Two reviewers, working independently, screened all titles and abstracts that were identified through the literature search. Furthermore, they selected potential studies by obtaining the full-text articles, and then evaluated them, in accordance with the eligibility criteria.

### Data extraction and risk-of-bias assessment

Two reviewers independently screened all the potential quantitative results or critical data from some preselected studies, with regard to the participants, interventions, control conditions, outcome measurements and results. Subsequently, disagreements between the reviewers were discussed with the ninth author, in order to reach a consensus.

The reviewers independently assessed risk of bias by using a version of the Cochrane Collaboration’s tool for assessing risk of bias.^14^ This includes nine domains: adequacy of sequence generation; allocation sequence concealment; blinding of participants and caregivers; blinding of data collectors; blinding for outcome assessment; blinding of data analysts; incomplete outcome data; selective outcome reporting; and presence of other potential sources of bias not accounted for in the previously cited domains. For incomplete outcome data, we stipulated that low risk of bias consisted of loss to follow-up of less than 10% and a difference in missing data between the intervention and control groups of less than 5%.

### Certainty of evidence

The reviewers used the Grading of Recommendations for Assessment, Development and Evaluation (GRADE) methodology to rate the certainty of scientific evidence for each outcome, which was categorized as high, moderate, low or very low.^15^ The GRADE approach assessed the following: overall risk of bias,^16^ imprecision,^17^ inconsistency,^18^ indirectness^19^ and publication bias.^20^ Thus, the results were summarized in an evidence table, i.e. as a GRADE evidence profile. 

The reviewers independently assessed eligibility, risk of bias and data abstraction. Disagreements were resolved by reaching a consensus or by obtaining a third reviewer’s opinion if needed.

### Data synthesis and statistical analysis

We pooled the data to calculate pooled risk ratios (RRs) or mean differences, with 95% confidence intervals (CIs), using a fixed-effect model by considering the last follow-up outcome that had been measured in each study included. We assessed heterogeneity by means of the I^2^ statistic and evaluated the quality of the evidence by using the GRADE method. All of the analyses were conducted using the Review Manager (RevMan) software.^21^

## RESULTS

### Selection of titles

**Figure 1:**
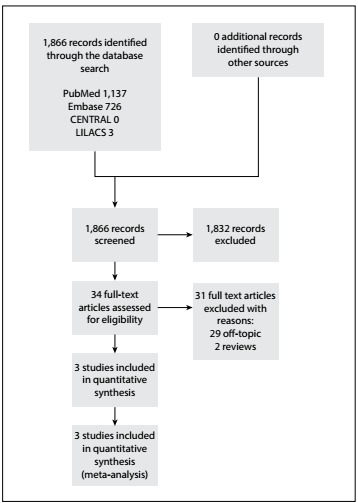
Flowchart for inclusion and exclusion of studies.

A total of 1,866 titles were identified in the databases cited above, but only 34 studies were selected for detailed evaluation.^12^ Ultimately, it was found that only three studies that included 343 patients were eligible for the current review (Figure 1).

These studies presented different interventions, i.e. distant reiki,^9^ regular reiki^10^ and prayer meditation,^11^ but they all presented a similar outcome, i.e. they measured pain through a visual analogue scale (VAS) and also measured heart rate and diastolic and systolic blood pressure. In the literature, all reiki healers consider that distant reiki^9^ and regular reiki^10^ are the same, with the only difference that in one, the patient`s physical body is absent, while it is present in the other.^1^^,^^2^^,^^9^

Although prayer meditation^11^ may seem to have been the odd one out, we analyzed this study in depth and decided to plot it together with the two reiki studies,^9^^,^^10^ because all the information from this study with regard to the prayer meditation background, the objectives of the study and the methods used to evaluate the intervention were in line with these other studies.^9^^,^^10^ Moreover, these factors were in line with our aim in this systematic review, which was to evaluate perceived pain among women undergoing cesarean section. Moreover, both reiki and prayer meditation are non-invasive and non-pharmacological practices, and both of them can be considered to be spiritual interventions.^1^^,^^2^^,^^9^^,^^10^^,^^11^

### Study characteristics

Table 2 describes the characteristics of the studies relating to their designs, settings, numbers of participants, interventions and usual care treatments received by the patients; and according to the hospital protocol, mean age, inclusion and exclusion criteria and follow-up after caesarean section. One study was conducted in Canada,^9^ and the other two were conducted in the Middle East, in Turkey^10^ and Iran.^11^ The sample sizes ranged from 40^9^ to 80^11^ pregnant women aged in their twenties or thirties. All the studies included pregnant women undergoing cesarean section.

**Table 2:**
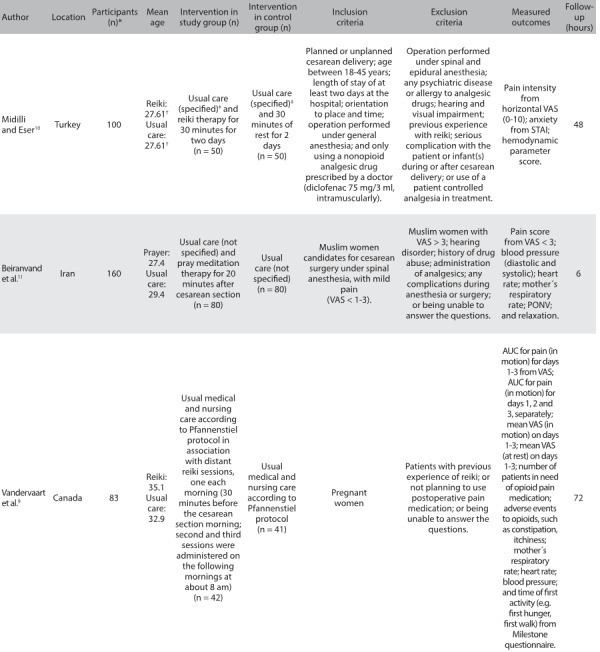
Study characteristics relating to setting, number of participants, mean age, intervention and control groups, inclusion and exclusion criteria, assessed outcomes and follow-up after caesarean section

n: number; RCT: randomized controlled trial; ml: milliliter, mg: milligram; VAS: visual analogue scale; STAI: State-Trait Anxiety Inventory; cm: centimeters; PONV: incidence of postoperative nausea and vomiting. *Randomized participants; ^†^Patients were equalized according to age into two groups (18-31 and 32-45 years old); ^‡^Day 1 (24 hours after operation): Patients were given the first dose of the standard analgesic (intramuscularly, 75 mg/3 ml diclofenac at 9.00 a.m. and 9.00 p.m.); Day 2 (48 hours after operation): Patients were given the third dose of the standard analgesic (intramuscularly, 75 mg/3 ml diclofenac at 9.00 a.m. and 9.00 p.m.); Day 3 (72 hours after operation): Minoset® 500 mg, tablet (every 4-6 hours, as needed) was administered in accordance with the analgesic protocol. At 72 hours only, the number of analgesics required by the patient was determined by means of face-to-face interview or telephone call.

The following exclusions of patients were made in one or more of these studies: previous experience with reiki;^9^^,^^10^ perception of pain on a visual analogue scale (VAS) > 3;^11^ not planning to use standard postoperative pain medication;^10^^,^^11^ not being able to answer the questions;^9^^,^^10^^,^^11^ visual and hearing impairment;^10^^,^^11^ any complications during anesthesia or surgery;^10^^,^^11^ history of drug abuse;^10^^,^^11^ operation performed under spinal and epidural anesthesia;^10^ use of patient-controlled analgesia in the treatment;^10^ and presence of any psychiatric disease or allergy to analgesic drugs.^10^ The length of the follow-up ranged from 6 hours to 3 days.

### Risk-of-bias assessment

**Figure 2:**
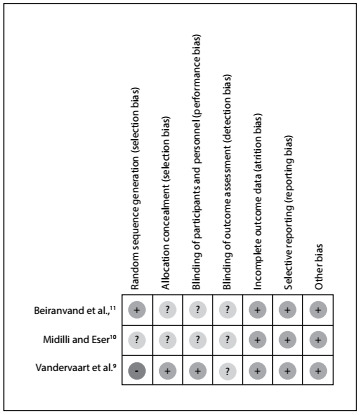
Risk-of-bias assessment.

Figure 2 describes the risk-of-bias assessment for RCTs. The overall methodological quality of the studies examined was evenly separated into unclear and low risk-of-bias categories. However, the main concern was the risk of bias relating to random sequence generation in the study by Vandervaart et al.^9^ Additionally, the allocation concealment and blinding of participants/personnel were uncertain in the studies by Midilli and Eser^10^ and Beiranvand et al.^11^ Finally, none of the three studies^9^^,^^10^^,^^11^ showed any certainty with regard to blinding of the outcome assessment.

### Effects of reiki and prayer meditation: meta-analysis

#### Pain score

**Figure 3:**
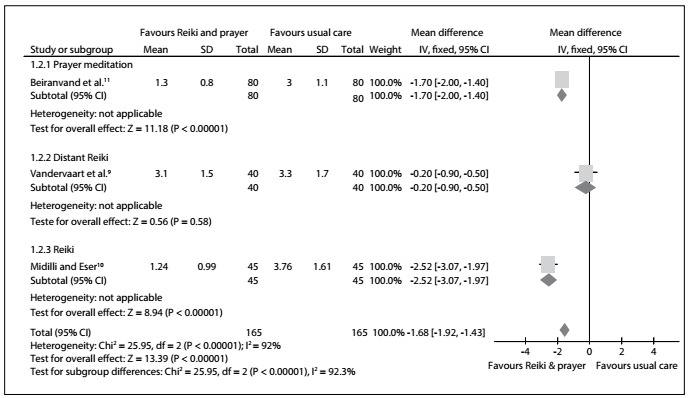
Meta-analysis on mean pain score measured using visual analogue scale (VAS).

Regarding the pain scores measured by means of a VAS in the overall analysis, the results from three RCTs^9^^,^^10^^,^^11^ found a statistically significant difference favoring reiki and prayer meditation over the usual care: mean difference (MD) = -1.68; 95% confidence interval (CI): -1.92 to -1.43; P < 0.00001; I^2^ = 92%. In relation to the following subcategories, we also found statistically significant differences favoring the integrative practices over the usual care: prayer meditation (MD = -1.70; 95% CI: -2.00 to -1.40; P < 0.00001; I^2^ = not applicable); and reiki (MD = -2.52; 95% CI: -3.07 to -1.97; P < 0.00001; I^2^ = not applicable). However, there was no statistically significant difference between the distant and regular reiki groups: MD = -0.20; 95% CI: -0.90 to 0.50; P = 0.58; I^2^ = not applicable. The certainty of the evidence was downrated to low because of inconsistency and publication bias (Figure 3, Table 3).

**Table 3:**
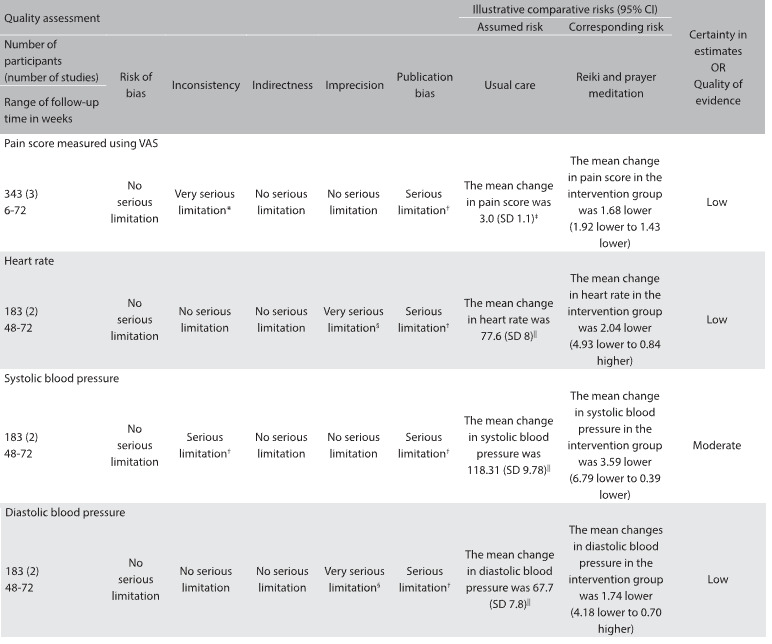
GRADE evidence profile for continuous outcomes: complementary alternative medicine for cesarean section

CAM = complementary alternative medicine; SD = standard deviation; std. = standardized; VAS = visual analogue scale. ^*^There was substantial heterogeneity (I^2^ = 92.3%) among the different interventions used in the studies included (i.e. distant reiki; reiki; and prayer meditation); ^†^There was heterogeneity (I^2^ = 26%) among the different interventions used in the studies included (i.e. distant reiki; reiki; and prayer meditation). ^‡^Baseline risk estimates for pain come from control arm of study by Beiranvand et al.^11^ (largest randomized trial in the meta-analysis); ^§^95% CI for absolute effects include benefit and harm. ^||^Baseline risk estimates for heart rate, systolic and diastolic blood pressure come from control arm of study by Midilli et al.^10^ (largest randomized trial in the meta analysis).

### Heart rate

**Figure 4:**
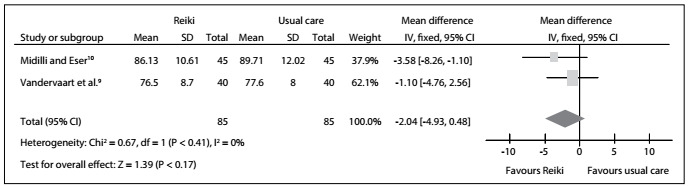
Meta-analysis on heart rate.

With regard to heart rate in the overall analysis, the results from two RCTs^9^^,^^10^ did not show any statistically significant difference that favored regular reiki and distant reiki over the usual care: MD = -2.04; 95% CI: -4.93 to 0.84; P = 0.41; I^2^ = 0%. Therefore, we found no statistically significant difference favoring reiki over the usual care: MD = -3.58; 95% CI: -8.26 to 1.10; P = 0.17; I^2^ = not applicable. In addition, there was no statistically significant difference between the distant and regular reiki groups: MD = -1.10; 95% CI: -4.76 to 2.56; P = 0.17; I^2^ = not applicable. The certainty of the evidence was downrated to low because of imprecision and publication bias (Figure 4, Table 3).

### Diastolic blood pressure

**Figure 5:**
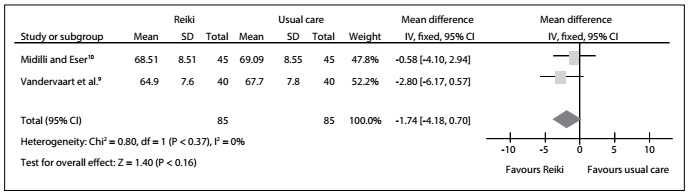
Meta-analysis on diastolic blood pressure.

For diastolic blood pressure, the results from two RCTs^9^^,^^10^ did not show any statistically significant difference favoring regular reiki and distant reiki over the usual care: MD = -1.74; 95% CI: -4.18 to 0.70; P = 0.16; I^2^ = 0%. Therefore, we also found no statistically significant difference favoring reiki over the usual care: MD = -0.58; 95% CI: -4.10 to 2.94; P = 0.37; I^2^ = not applicable. In addition, there was no statistically significant difference between the distant and regular reiki groups: MD = -2.80; 95% CI: -6.17 to 0.57; P = 0.37; I^2^ = not applicable. The certainty of the evidence was downrated to low because of imprecision and publication bias (Figure 5, Table 3).

### Systolic blood pressure

**Figure 6:**
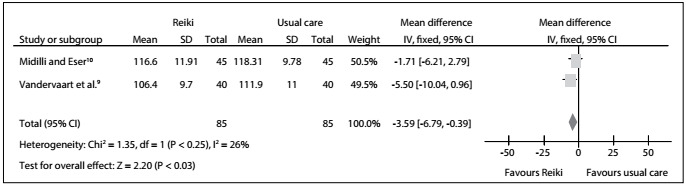
Meta-analysis on systolic blood pressure.

In the overall analysis with regard to systolic blood pressure, the results from two RCTs^9^^,^^10^ showed that there was no statistically significant difference favoring reiki over the usual care: MD = -3.59; 95% CI: -6.79 to 0.39; P = 0.03; I^2^ = 26%. Therefore, we also found no statistically significant difference favoring reiki over the usual care: MD = -1.71; 95% CI: -6.21 to 2.79; P = 0.25; I^2^ = not applicable. In addition, there was no statistically significant difference between the distant reiki and regular reiki groups: MD = -5.50; 95% CI: -10.04 to -0.96 P = 0.25; I^2^ = not applicable. The certainty of the evidence was downrated to moderate because of inconsistency and publication bias (Figure 6, Table 3).

### Effect of first-time activity through the Milestone questionnaire and patients’ need for opioids in the Vandervaart study^9^

Regarding the activity milestone questionnaire, which is used among women after elective caesarean to evaluate the rate of healing, a single RCT^9^ showed that there was no statistically significant difference between distant reiki and the usual care in any of the following categories: time to first hunger; time to first eating of solid food; time to first flatus; time to first bowel movement; time to first spontaneous voiding; and time to first ambulation (Figure 1). Moreover, the same study^9^ described the patients’ need for opioids, but showed that there was no statistically significant difference between distant reiki and the usual care on the day of admission to hospital (relative risk, RR = 0.81; 95% CI: 0.66 to 1.30; P = 0.64; I^2^ = not applicable); or on the next day (RR = 1.22; 95% CI: 0.74 to 1.63; P = 0.65; I^2^ = not applicable) (Figure 2).

## DISCUSSION

This study evaluated the use of reiki and prayer meditation for pain control among women undergoing caesarean section. It was the first-ever study to evaluate spiritual healing in relation to this issue, given that most previous studies and reviews focused on severe chronically ill patients and their quality of life.^1^^,^^2^^,^^3^^,^^4^^,^^5^^,^^6^^,^^7^

It should be noted that a language restriction had to be imposed during the selection process due to lack of funds to pay for translation services prior to the search. Thus, it was necessary to restrict the current systematic review to English and Portuguese-language studies were assessed from the outset. However, no study was excluded because it was written in another language, because no such studies were found through the search methods.

A recent study^22^ showed that around 26% of women in the United States received spiritual healing treatment if they were non-smokers, non-drinkers or low-risk drinkers, had symptoms of severe tiredness, depression, anxiety, diagnosed cancer or major illnesses. In another study on women in the southern and midwestern United States regions (i.e. extremely religious areas) the proportion that received prayers for health was estimated to be 53%.^23^ The fact that the study by Beiranvand et al.^11^ presented a significant outcome, i.e. pain control among women undergoing caesarean sections, with high follow-up rates, may have been due the presence of highly religious women in their sample. According to Bell et al.,^23^ people who usually use prayer meditation are also likely to use some other form of complementary or alternative medicine (e.g. reiki or acupuncture). Additionally, the more religious the people are, the more likely it is that they will use preventive healthcare.^20^

Among the studies included, some limitations were reported, as follows: the sample size;^9^^,^^10^^,^^11^presence of only one reiki therapist;^9^ absence of information about the mechanism of action of distant reiki;^9^ use of shared rooms;^10^ and a noisy environment.^10^ In addition to the methodological limitations, this current review also presented a limitation relating to the results obtained through meta-analysis: although the perceived pain seemed to have decreased significantly, the heterogeneity of results was extremely high, i.e. I^2^ = 92% (Figure 3). This can be explained by the fact that there were three different types of intervention.

On the other hand, regular and distant reiki work in the same way as foundations for this type of therapy^9^ and, therefore, they were not different at all. Moreover, prayer meditation is a form of adjunctive therapy within many cultures.^11^^,^^20^^,^^23^^,^^24^ Thus, both prayer meditation and reiki are forms of spiritual healing. According to Benor,^24^ spiritual healing is defined as a systematic and purposeful intervention by practitioners that has the aim of helping other people to improve their health condition through focused intention, which can include hand contact or hand movement. Thus, these three studies^9^^,^^10^^,^^11^presenting similar methodological aspects and outcomes were plotted together. Within systematic reviews, it is known that meta-analyses that included less than 10 studies cannot to estimate heterogeneity.

The meta-analysis did not show any statistical significant differences from before to after the treatments, either in the intervention or in the usual care group, regarding heart rate (Figure 4), diastolic and systolic blood pressure (Figures 5 and 6, respectively). In other words, these results were concordant with those of the previous review.^2^ However, to reach definitive conclusions regarding the effectiveness of such therapies, larger populations in good RCTs are needed.

With regard to the methodological aspects, the present review noted that there were risks of bias relating to random sequence generation;^9^ allocation concealment;^10^^,^^11^ blinding of participants/personnel;^10^^,^^11^ and blinding of outcome assessment. This concern corroborates what was reported in the systematic review on reiki conducted by Vandervaart:^2^ all of the 12 studies included had failings in at least in one of the following areas: randomization, blinding and accountability of all patients. Therefore, both reviews can be classified as presenting low-quality evidence, and the main issue in this regard is the poor evidence from the RCTs. We sent emails to the respective corresponding authors of the studies selected for this review,^9^^,^^10^^,^^11^ regarding points in these studies that were unclear to us or not reported, but no replies had been received by the time of submitting this review.

The previous review^2^ attempted to evaluate the effectiveness of reiki therapy under several conditions and presented 31 different outcomes within the 12 studies included. Not all of these studies were RCTs; no meta-analysis was performed, and the findings were based on Jadad scores. The previous review also did not include any study on pregnant women undergoing a cesarean section. On the other hand, the present systematic review included three studies^9^^,^^10^^,^^11^ in which there were similarities regarding methods, outcomes and populations, based our evidence from the GRADE profile for continuous outcomes (Table 3), and this review also included a meta-analysis.

Additionally, the major limitation of the current study was that only a very small number of studies considering spiritual healing approaches to pain management after cesarean section have been published. Therefore, there is still a need for high-quality RCTs on this issue, with the aim of assessing the real effectiveness of reiki and prayer meditation in relation to pain control among women undergoing cesarean section.

## CONCLUSION

Low-certainty evidence suggested that use of reiki and prayer meditation might be associated with pain reduction.
